# A Pilot Study of Baseline Spatial Genomic Heterogeneity in Primary Gastric Cancers Using Multi-Region Endoscopic Sampling

**DOI:** 10.3389/fonc.2020.00225

**Published:** 2020-02-25

**Authors:** Joseph Chao, Jeeyun Lee, Kyung Kim, So Young Kang, Taehyang Lee, Kyoung-Mee Kim, Seung Tae Kim, Samuel J. Klempner, Hyuk Lee

**Affiliations:** ^1^Department of Medical Oncology and Therapeutics Research, City of Hope Comprehensive Cancer Center, Duarte, CA, United States; ^2^Division of Hematology-Oncology, Department of Medicine, Samsung Medical Center, Sungkyunkwan University School of Medicine, Seoul, South Korea; ^3^Department of Pathology and Translational Genomics, Samsung Medical Center, Sungkyunkwan University School of Medicine, Seoul, South Korea; ^4^Department of Medicine, Massachusetts General Hospital Cancer Center, Boston, MA, United States; ^5^Harvard Medical School, Boston, MA, United States; ^6^Division of Gastroenterology, Department of Medicine, Samsung Medical Center, Sungkyunkwan University School of Medicine, Seoul, South Korea

**Keywords:** gastric cancer, intratumoral heterogeneity, endoscopy, precision medicine, circulating tumor DNA

## Abstract

Intertumoral heterogeneity among actionable biomarkers including *ERBB2, FGFR2* and *EGFR* has been observed to occur under therapeutic pressure in advanced gastric cancer. However, baseline intratumoral heterogeneity at diagnosis is understudied and may impact clinical outcomes. We sought to explore intratumoral heterogeneity in primary advanced gastric cancers via DNA sequencing from multi-region endoscopic sampling at diagnosis. Patients with newly diagnosed advanced gastric adenocarcinoma underwent endoscopic mapping and pre-determined 8-sector biopsy of the primary tumor with concurrent plasma cfDNA sampling. Biopsy samples were subjected to targeted next generation sequencing and plasma cfDNA was analyzed via a 28-gene cfDNA assay. Expectedly, we observed that the majority of genetic alterations were shared among multi-sector biopsies within the same gastric primary tumor. However, all samples contained private subclonal alterations between biopsy sectors, including actionable alterations in *GNAS* and *STK11*. Cell free DNA analyses also exhibited both shared and non-shared alterations between mutations detected in cfDNA and tumor tissue biopsies confirming baseline intertumoral heterogeneity. This is the first dataset to confirm baseline intratumoral heterogeneity and confirms that multi-sector endoscopic biopsy is feasible and capable of capturing intratumoral heterogeneity among relevant genomic alterations in gastric cancer. Both multi-sector endoscopic biopsies and cfDNA analyses are complementary in capturing the diverse mutational landscape at disease presentation.

## Introduction

Treatment options for gastric and gastroesophageal junction (GEJ) cancers have continually evolved with improved understanding of the diverse molecular landscape stemming from large scale next generation sequencing efforts (1, 2). An appreciation of inter-patient tumoral heterogeneity has necessitated multiple biomarker testing to provide maximal predictive and prognostic information. Despite limited data, panel-based sequencing approaches have entered into clinical practice for gastric cancer allowing for simultaneous determination of actionable biomarkers including microsatellite instability (MSI) and expression of HER2 and PD-L1 to guide approved biologic therapies. However, reliable determination of tumor biomarker status is hindered by the recognition that gastric cancer intratumoral heterogeneity exists, consistent with the theory that cancers should be viewed as macro-evolutionary clonal populations (3). HER2, since its approval as a validated biomarker for the monoclonal antibody trastuzumab, is well-known to exhibit intratumoral heterogeneity, exemplified by case series reporting differences in positivity rates from repeated endoscopic sampling (4–6). Large scale comprehensive molecular analyses from The Cancer Genome Atlas (TCGA) and Asian Cancer Research Group (ACRG) have yielded other genomic alterations that can serve as attractive targets for molecularly targeted therapies (1, 2). However, the study of intratumoral heterogeneity still remains in its relative infancy, with analyses of primary tumors and synchronous metastatic sites providing proof of principle that heterogeneity impacts targeted therapy outcomes (7–11). Further hindrance to the study of intratumoral heterogeneity for gastroesophageal cancer is standard endoscopic biopsies typically have limited sampling of the primary tumor. With HER2 being the prototypical gastric cancer biomarker, studies have suggested increasing endoscopic sampling of ≥4 biopsy fragments can increase the sensitivity of detecting tumor HER2 overexpression (12). Advent of next generation sequencing (NGS) has enabled detailed genomic study of clonal evolution events and development of intratumoral heterogeneity across multiple malignancies (13–15). As such, we conducted an initial study implementing systematic multi-spatial endoscopic sampling of gastroesophageal tumors paired with downstream NGS in attempts to characterize intratumoral heterogeneity of oncogenic alterations beyond HER2.

## Results

### Patients and Multi-Region Biopsy Feasibility

**Figure 1:**
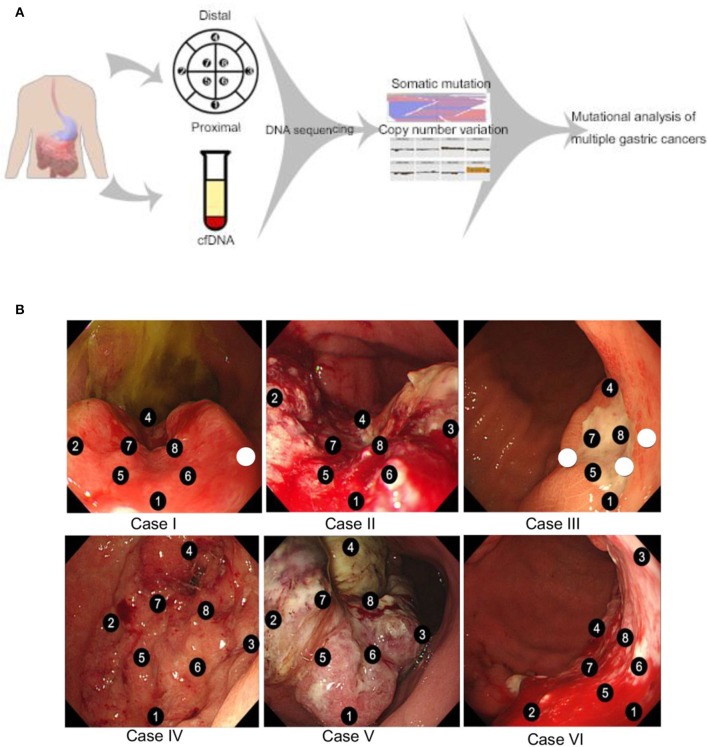
**(A)** Schematic overview of the study design. Endoscopic mapping was preplanned for eight sectors of the primary tumor with proximal and distal orientation. Tumor tissue somatic mutations and copy number variants were determined by targeted next generation sequencing along with cell free DNA analysis. **(B)** Endoscopic mapping for multiple biopsy. Eight specimens were obtained from each patient case. Sectors with insufficient purity of tumor DNA for next generation sequencing are represented by unnumbered circles.

Eight patients were enrolled to this tissue sampling study with pre-specified 8 sector mapping and sampling (Figure 1A). Data from 2 patients were excluded from the analysis since <4 pieces (of eight biopsied specimens) passed QC. All six patients presented with metastatic disease at study enrollment with representative clinicopathologic characteristics (Table 1). Two patients were missing 1 (Case I) or 3 sector(s) (Case III) of NGS results due to insufficient tumor DNA purity from a given biopsy location (white circle in Figure 1B). Overall, we obtained 44/48 (91.6%) of the intended biopsies supporting the feasibility of our endoscopic mapping approach. Only a single inner quadrant biopsy (location 6 in case III) was insufficient for downstream analysis whereas three outer quadrant biopsies contained insufficient tumoral DNA for sequencing. Notably, case III is a young woman with diffuse type signet ring cell gastric cancer, a subset enriched for the TCGA genomically stable subtype which may explain lower tumoral cellularity and DNA content (1).

**Table 1:** Clinicopathologic characteristics of the gastric cancer study population.

**Case #**	**Age at diagnosis**	**Sex**	**Pathology**	**Lauren classification**	**Stage**	**Metastatic sites**
Case I	78	M	Adenocarcinoma—poorly differentiated	Diffuse	IV	Left adrenal, Distant lymph nodes
Case II	57	M	Adenocarcinoma—moderately differentiated	Intestinal	IV	Omental seeding
Case III	21	F	Signet ring cell adenocarcinoma	Diffuse	IV	Multiple bone metastases
Case IV	60	M	Adenocarcinoma—poorly differentiated	Indeterminate	IV	Multiple distant lymph nodes
Case V	78	M	Adenocarcinoma—poorly differentiated	Indeterminate	IV	Multiple distant lymph nodes
Case VI	56	M	Adenocarcinoma—moderately differentiated	Indeterminate	IV	Multiple distant lymph nodes, peritoneal carcinomatosis

### Inner and Out Region Primary Tumor Biopsies Demonstrate Intratumoral Heterogeneity

**Figure 2:**
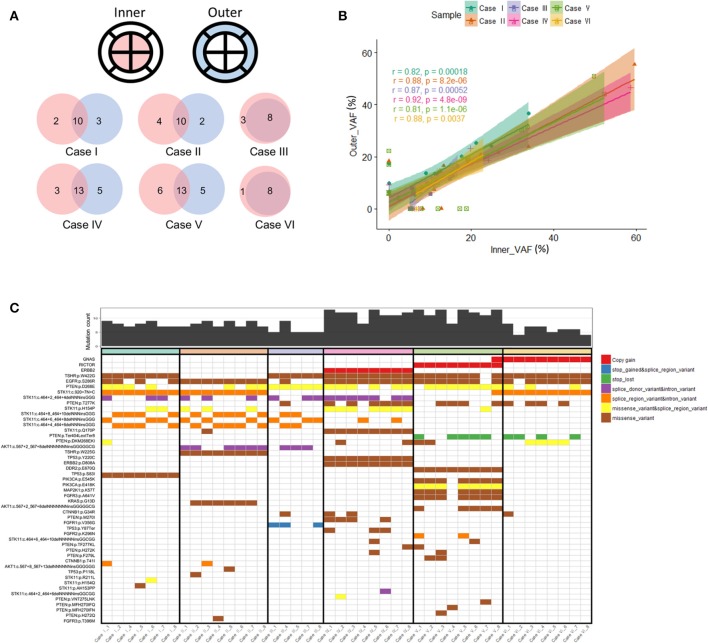
**(A)** Venn diagram illustrating overlapping somatic mutations detected in inner vs. outer biopsies. In all six cases more than half of mutations were shared between inner and outer biopsies. **(B)** Correlation coefficients of variant allele frequencies (VAFs) between inner and outer biopsies. The plot is representative of VAFs of identified SNVs and INDELs among the six cases. Mutations falling within amplified genes were not considered in the correlation analysis. The Pearson correlation coefficient between the variants from inner and outer biopsies on average was 0.81 or more. **(C)** Genomic landscape of mutations detected among all analyzable biopsies. In total 48 unique alterations were identified amongst 17 genes with evidence of both shared and non-shared mutations in the differing biopsies of the same primary tumor.

We sought to compare inner and outer quadrant biopsies to interrogate intratumoral heterogeneity in the primary tumor prior to any therapy. As expected, inner and outer quadrant biopsies demonstrated a majority of shared genomic alterations (Figure 2A). Overlap was a predominant feature such that across all identified variant allele frequencies a statistically significant positive linear correlation was observed between single-nucleotide variant (SNV) and insertion-deletion (INDEL) mutations from inner and outer sector biopsies (Figure 2B). This is consistent with observations from primary-metastasis paired whole exome analyses in gastroesophageal cancers. Importantly, all samples also contained some alterations that were private to either the inner or outer quadrants (Figures 2A–C). Within our dataset the number of shared alterations within a given case was significantly lower than the number of non-shared alterations (*p* = 0.004). However, the average mutant allele frequency was significantly higher among shared alterations within a case vs. the non-shared alterations (*p* = 0.009). Within a class of genomic alteration only amplifications (*ERBB2, RICTOR, GNAS*) in cases 4–6 were concordant across all multi-region samples (Figure 2C). Private subclonal SNV and INDELs, including pathogenic *CTNNB1* and *PTEN* mutations, existed in 6/6 (100%) of our samples, also consistent with prior multiregion sequencing in other tumor types (16–21).

Case V highlights the putative clinical implications of baseline intratumoral heterogeneity and harbored a non-shared *GNAS* amplification detected in only one of the eight sectors of the primary tumor (Figure 2C). *GNAS* encodes a G protein alpha stimulatory subunit and is of interest given activating mutations have been proposed to mediate resistance to EGFR inhibitors and activate Wnt/β-catenin signaling pathways in gastric adenocarcinomas (22, 23).

### Cell-Free DNA Confirms Baseline Intertumoral Heterogeneity in Untreated Gastric Cancer

**Figure 3:**
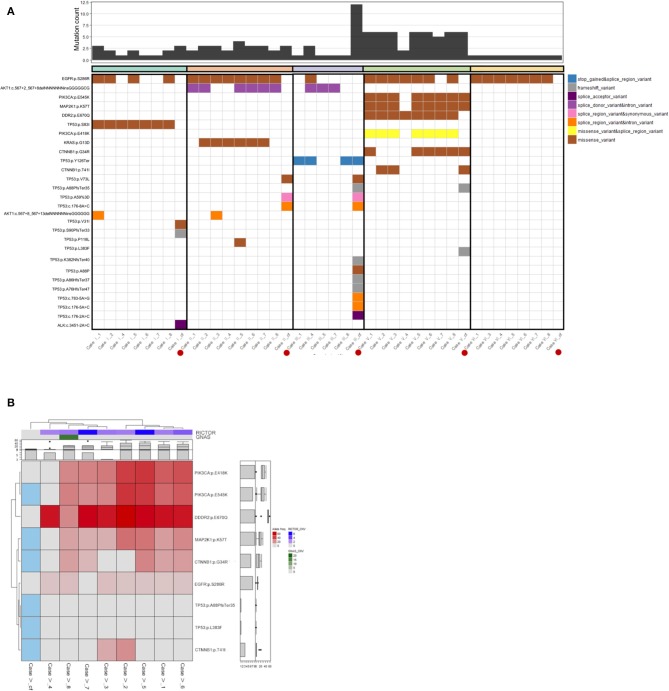
**(A)** Genomic landscape of mutations detectable by both the Archer solid tumor and cfDNA assay. We focused on the 20 genes common to both assays to analyze mutational heterogeneity from endoscopic multi-sector tissue sampling and cfDNA. Case III represented a case with the greatest number of non-shared mutations detected in cfDNA but not tumor tissue, while Case VI was representative of a case with no detectable cfDNA alterations. **(B)** Heat map of detected gene mutations in Case V. The mutational allele fraction of genes with detectable alterations from the Archer solid tumor panel were standardized and represented as a heat map. For cfDNA, detection was dichotomously represented, with Blue indicating detection, and Gray indicating no detection. Case V among the six cases was representative of the greatest number of shared alterations between the solid tumor panel and cfDNA testing.

We conducted cfDNA sequencing from concordant blood samples collected from our six cases to investigate how circulating tumor DNA profiling may reflect intratumoral and intertumoral heterogeneity. Whole blood (10 mL) was taken immediately prior to planned endoscopy to minimize confounding cfDNA that may be shed from biopsy sampling. We focused our analysis to the 20 genes (Supplementary Table 1) common to both the Archer solid tumor and cfDNA assays (Figure 3A). Genes included in both tissue and cfDNA sequencing included multiple known to be important and potentially actionable in gastric cancer including *ERBB2* (10), *CTNNB1* (24), *EGFR* (22), *MET* (7), and *KRAS* (25). In each case with detectable or available cfDNA we observed cfDNA-detected alterations not observed in concurrent tissue sequencing, supporting pre-treatment inter-tumoral heterogeneity.

In terms of number of detectable unique cfDNA alterations for each case, they ranged from 0 (Case VI) to 12 mutations (Case III). Interestingly for Case III, whose clinical presentation was that of multiple bony metastases, 11 of the 12 cfDNA alterations were non-shared with any of the tissue biopsy results, and were represented by a collection of frameshift and splice variant mutations in the p53 gene. The only common alteration captured in 3 of the 5 analyzable tissue biopsy sectors that was also detectable in cfDNA was a *p53* p.Y126Ter mutation leading to a truncated gene product. Given the vast majority of *p53* cfDNA mutations occurred proximal to codon 126, this observation could support these mutations exist in *cis* or *trans* and these subclones exist at a very low proportion within the primary tumor. The alternative, and more likely conclusion, is these p53 mutations are representative of circulating tumor DNA shedding from metastatic clonal populations, though germline single nucleotide polymorphisms cannot be completely ruled out by our methods. In Case V we observed the greatest number of shared alterations between tumor tissue and cfDNA represented by four gene mutations (*PIK3CA* p. E545K, *MAP2K1* p. K57T, *CTNNB1* p.G34R, and *CTNNB1* p. T41I) (Figure 3B). Also for Case V, sufficient samples remained to subject four of the eight biopsied sectors to NGS utilizing an independent gene assay (Oncomine Comprehensive Assay v3). Interestingly, a missense mutation in *DDR2* (p. E670Q), which was detected at high MAF (>40%) across nearly every endoscopic biopsy sector, was not detected in cfDNA. This *DDR2* mutation was also validated with the Oncomine panel at high MAF in the 4 sectors with sufficient tumor tissue remaining for reanalysis. Furthermore, we also observed *PIK3CA* (p. E418K) and *EGFR* (p. S286R) alterations that were detected in seven of eight endoscopic biopsy sectors, albeit at relatively lower MAF (>20 and >5%, respectively), that were not represented in cfDNA. Validation utilizing the Oncomine assay confirmed presence of the *PIK3CA* (p. E418K) mutation in the four available sectors, though the *EGFR* (p.286R) alteration was not detected on reanalysis of the four regions possibly accounted for by the low MAF of the latter.

### High Frequency of STK11 Alterations Observed in Combined Tumoral and cfDNA Analysis

Our dataset also exhibited a high proportion of alterations in the *STK11* tumor suppressor gene, something that was unexpected. Within the gastric cancer TCGA (*n* = 393 samples with sequencing and copy-number data) the frequency of *STK11* alterations is 4% (www.cbioportal.org, data not shown). Although Asians are underrepresented in the TCGA, which is almost entirely non-metastatic surgical samples, 4/14 *STK11*-altered samples (29%) are from Asian patients. Although severely limited by small sample size (*n* = 6) we observed at least one *STK11* alteration in each tissue sample (Figure 1C). Larger Asian series from advanced gastric cancer patients will be needed to examine whether or not *STK11* alteration frequencies differ between Western and Asian populations.

Inactivating *STK11* mutations have recently emerged as a putative mechanism of resistance to PD-1 inhibitors in non-small cell lung cancer (NSCLC) (26, 27). A shared *STK11* DNA coding position 920 splice variant mutation was detected in all analyzable sectors for Cases I, II, and VI, while an *STK11* p. Q170P mutation was detected in all eight sectors for Case IV. Among non-shared *STK11* alterations we made some interesting observations. In Case I, additional missense mutations for *STK11* were detected in only one of seven sectors (1 each p. AH153PP and p. H154Q) or two of seven sectors (p. H154P). In Case II we also made a similar observation of additional *STK11* missense mutations being detected in only one of eight sectors (1 each p. Q170P and p. R211L) or three of eight sectors (p. H154P). Presumably these are representative of subclonal tumor cell populations with the *STK11* splice variant mutation detected in all sectors representing a truncal mutation. In Case V we did not observe *STK11* mutations being a shared alteration across multiple biopsy sectors. Of note, there was the detection of an *STK11* missense mutation (p. H154P) in only one of the inner biopsy sectors. The mutation allele frequency (MAF) for this alteration was 22.3%, implying this was a notable subclone spatially localized within one region of the primary gastroesophageal tumor. Unfortunately, no further patient sample remained from this biopsy sector to validate the restricted localization of this specific STK11 subclone. Arguably, this still represents a potentially clinically relevant mutation that may have been easily missed with limited endoscopic sampling of this primary tumor.

## Discussion

Here we provide the first multi-region next-generation sequencing from primary gastric tumors in a cohort of treatment naïve stage IV patients and confirm baseline spatial intratumoral and intertumoral heterogeneity with cfDNA. Our findings support the emerging data that tumoral heterogeneity represents a barrier to targeted approaches in gastric cancer (16–21, 24, 28). In addition, the variation of genetic alterations in differing regions of a primary gastric cancer adds to the literature of caution needed in informing clinical treatment decisions from single gene or NGS analyses of small biopsy samples in which a biomarker may be absent from a biopsy region (29). The interrogation of circulating tumor DNA more reflective of the global genomic tumor landscape can overcome some limitations from tissue sampling assays. Our work, and several prior reports demonstrate the complementary nature of cfDNA in identifying subclonal private alterations representing inter-tumoral heterogeneity (10, 20, 29). The presence of detectable cfDNA mutations (such as in *TP53*) not being represented in tumor supports a role for cfDNA whereas our findings of genomic alterations (including *PIK3CA* and *EGFR*) being detected robustly in tumor tissue sampling but not represented in cfDNA fractions cautions against complete abandonment of tumor tissue sequencing. Multiple orthogonal methods will complement each other in obtaining the full genomic footprint associated with a gastric cancer presentation, and other -omic platforms will provide further data. Judicious implementation of tissue collection protocols may provide sufficient biologic and technical replicates to overcome sensitivity and specificity limitations of NGS assays. The establishment of intratumoral heterogeneity has generally been attributed to gradual mutations over time selecting for favorable subclones that may coexist in the total composition of a growing tumor (30, 31). Other studies also support a single catastrophic genome-wide mutational and chromosomal rearrangement event (chromothripsis) occurring and subsequently driving the outgrowth of a selectively favorable clone (32–34). The understanding of such spatial and temporal mechanisms will be integral to novel therapeutic strategies in advanced gastroesophageal cancer as there are multiple models of metastatic spread (35). Inevitably drug resistance develops with current treatment approaches and identification of new molecular drivers in cfDNA may represent selection of subclones that were invariably present within the primary gastric tumor, an observation seen in kidney cancer and non-small cell lung cancer (16, 17). In fact, discordance in actionable GEA biomarkers including HER2, EGFR, and MET in advanced untreated patients is observed in nearly 1/3 of patients (24). Under therapeutic pressures, in particular with “targeted therapies” such as trastuzumab, emergence of adaptive resistance alterations including *EGFR* amplification and *CCNE1* amplification has been identified by cfDNA in advanced GEA (10). As we have focused on baseline assessment only, our series is not designed to study clonal selection over time, though this is the focus of other ongoing work.

Our current observations would support multi-region endoscopic sampling with currently available endoscopic approaches as a feasible method to capture the heterogeneity of tumor subclones at disease presentation (16, 17). In fact, there was not additional toxicity with multiregional biopsy and our yield of 92% for downstream analyses is favorable. We feel this is clinically important as pre-clinical data suggests that the degree of baseline spatial heterogeneity may impact time to both recurrence and innate resistance (36). With larger sample sizes and longer follow up one could envision a prognostic role for a “heterogeneity score” in advanced gastric cancer, perhaps even inclusion in staging (37).

Our analysis is understandably limited by the small number of patients included in this study and relatively small number of genes tested for alterations. However, we note that multiple prior series examining tumoral heterogeneity have utilized single patients or very small series (16–21, 38). Consistent with prior approaches we conducted multi-faceted sampling from each patient and chose an NGS platform with both solid tumor and cfDNA analysis capability to minimize SNV, Indel, and CNV detection variability that may be introduced with differing sequencing platforms. Although the optimal gene panel size providing a balance of actionable clinical information and cost-effectiveness is not known, we feel the commercially available and validated assays chosen provide important clinical information. Specifically, both panels included the established GEA biomarkers *ERBB2* (10), *CTNNB1* (24), *EGFR* (22), *MET* (7), and *KRAS* (25) allowing for assessment of baseline heterogeneity in clinically relevant genes. We attempted to validate our findings through a separate research NGS panel, but were limited by the quantity of biopsy specimens remaining though did observe confirmation of relevant alterations in one of our cases. We would expect that larger platforms including whole exome sequencing (WES) would show a similar pattern of shared and private alterations, as has been seen in other tumor types. The clinical utility of WES to refine heterogeneity assessment remains unknown.

We also are unable to address the question if biopsies restricted to the visualized surface of the primary tumor may miss identification of subclonal populations that reside only deep within the tumor. Complete surgical removal of the primary gastroesophageal tumor has certainly been shown by us and others to be feasible in describing gastric cancer spatial intratumoral heterogeneity from non-metastatic patients (9, 39). Ideally a complete dataset would be composed of multi-region endoscopic biopsy, cfDNA, and then complete surgical resection of the primary tumor with no intervening therapy to alter its genomic landscape. However, surgical removal is not a standard of care with metastatic disease presentation as recently reaffirmed by modern randomized trials (40). This would limit primary tumor heterogeneity studies within this clinical context which arguably has greater therapeutic need than the non-metastatic setting. The three dimensional architecture of intratumoral heterogeneity in gastric cancer still remains poorly understood, though some recent mathematical models would argue genetic events leading to favorable evolutionary selection should not limit the distribution of rapidly growing subclones to the center of tumors (41). As such pure tumor surface endoscopic sampling may be sufficient to capture intratumoral heterogeneity to inform novel investigational treatment strategies in the clinic. Three-dimensional endoscopic mapping may provide further refinements in intratumoral heterogeneity but is not standard and difficult to adapt broadly. However, our overall approach is consistent with accepted methods to study heterogeneity.

An interesting observation of unknown significance is the high rate of *STK11* alterations in our pilot study. As noted previously, this is discrepant with the 4% rate seen across the TCGA. Notably, our patient cohort is all Korean advanced gastric cancer, and nearly 30% of the *STK11*-altered TCGA samples are from Asian origin. Owing to small sample size we cannot draw further conclusions, though examining larger Asian cohorts may be warranted to examine whether or not *STK11* differs between Western and Asian gastric cancers. In light of the observed differences in outcomes of Asian and non-Asian populations in some gastric cancer trials international collaborations are critical. This current study represents initial forming of an international effort (the Trans-Pacific Partnership for the Study of Heterogeneity in Tumors).

In conclusion, we provide pilot evidence that endoscopic multi-region sampling combined with concurrent cfDNA is feasible and identifies baseline gastric cancer intratumoral and intertumoral molecular heterogeneity. Our results lend credence to prospective study in larger patient cohorts and incorporation in biomarker-driven interventional trials.

## Methods

### Study Population and Tissue Sampling

Patients with newly diagnosed gastric cancer at Samsung Medical Center were prospectively identified for tissue sampling under an IRB approved protocol. Clinicopathologic characteristics including age, sex, histologic subtype, primary location, and metastatic pattern were collected from patient charts. All cases examined were patients with a primary gastric cancer diagnosis and no history of previous malignancy nor known germline mutations related to hereditary cancer syndromes. To study spatial intratumoral heterogeneity, systematic multi-regional sampling of primary tumors was achieved via preplanned endoscopic mapping of up to 8 visualized sectors (Figure 1A). Concordant blood sampling before the day (D-7–D0) of endoscopy examination was also obtained to capture circulating tumor DNA characteristics at the time of endoscopic biopsies. This study was approved by the Institutional Review Board of the Samsung Medical Center (Number 2014-04-119), and all patients provided informed consent. If tumor content was estimated as more than 40% after pathological review, tumor DNA and RNA were extracted from freshly obtained tissues using a QIAampini Kit (Qiagen, Hilden, 389 Germany) according to the manufacturer's instructions. In cases with DNA, we used RNaseA (cat. #19101; Qiagen). We measured concentrations and 260/280 and 260/230 nm ratios with an ND1000 spectrophotometer (Nanodrop Technologies, Thermo-Fisher Scientific, MA, USA) and then further quantified DNA/RNA using a Qubit fluorometer (Life Technologies, CA, USA). Details from DNA extraction are presented in Supplementary Table 1. A patient was excluded from further analysis if <6 of the pre-planned eight multi-region biopsies met the QC parameters.

### Analysis of Tissue Somatic Mutation

The Archer® customized VariantPlex® assay was employed for targeted NGS detection of CNVs, SNVs, and indels across 32 genes (Supplementary Table 1). Briefly, DNA fragmentation, end repair, dA-tailing and adaptor ligation for library preparation was achieved by following manufacturer standardized protocol for Illumina (www.archerdx.com, online Supplemental Information). Target enrichment was achieved with Anchored Multiplex PCR (AMP™) using independent gene-specific primers and molecular barcoded adapters for open-ended amplification of genomic DNA fragments. VariantPlex CTL libraries produced were sequenced to a minimum of 1.0 M reads per sample. Somatic variants were identified and annotated using Archer Analysis 5.0.6 for point mutations, indels and CNVs, respectively. To obtain reliable and robust mutation calling, we performed visual inspection of reads and “somatic variant” filtered the (i) AO ≥ 5. (ii) AF ≥ 0.05. (iii) Consequence like “coding_sequence_variant,” “feature_elongation,” “feature_truncation,” “frameshift_variant,” “incomplete_terminal_codon_variant,” “inframe_deletion,” “inframe_insertion,” “missense_variant,” “protein_altering_variant,” “splice_acceptor_variant,” “splice_donor_variant,” “splice_region_variant,” “start_lost,” “stop_gained,” and “stop_lost.” (iv) Variant Call is not “.NO CALL” and “homozygous reference”. The “Somatic CNV” filtered was (i) Amplification fold change threshold ≥2.5. (ii) Deletion fold change threshold ≤ 0.3333. (iii) *p* < 0.01. Germline status was not interrogated. With patients where sufficient biopsy material remained samples were submitted for repeat NGS using a validated research panel. For library preparation, the multiplex PCR-based Ion Torrent AmpliSeqTM technology (Life Technologies, Thermo Fisher Scientific) with the Oncomine™ Comprehensive Assay v3 (IonTorrent, Thermo Fisher Scientific) was used. This panel was designed to amplify 2530 DNA amplicons covering 143 cancer-related genes (42). Library preparation was carried out using the Oncomine Assay™ [comprising the DNA Oncomine™ Focus Assay (Thermo Fisher Scientific) and RNA Oncomine™ Fusions assay (Thermo Fisher Scientific)] following manufacturer's instructions using a total of 10 ng input DNA.

### Analysis of cfDNA Somatic Mutation

Ten milliliters of whole blood was taken at the time of endoscopy (D-7–D0). The commercially available and validated Archer® Reveal ctDNA™ 28 Kit was utilized for targeted NGS of circulating cell-free tumor DNA (ccfDNA/cfDNA/ctDNA) from 28 genes (Supplementary Table 1). Library preparation followed the established Archer® Reveal ctDNA™ protocol, and target enrichment was achieved with Anchored Multiplex PCR (AMP™), which uses independent gene-specific primers and molecular barcoded adapters for open-ended amplification of genomic DNA fragments. Somatic variants were identified and annotated using Archer Analysis 5.1 for point mutations, indels and CNVs, respectively. Germline status is not investigated in this method.

## Data Availability Statement

The raw data supporting the conclusions of this article will be made available by the authors, without undue reservation, to any qualified researcher.

## Ethics Statement

All participants provided written informed consent, and this study was approved by the Institutional Review Board of the Samsung Medical Center. All procedures performed in studies involving human participants were in accordance with the ethical standards of the institutional and/or national research committee and with the 1964 Helsinki declaration and its later amendments or comparable ethical standards.

## Author Contributions

JC, JL, KK, SJK, HL: conception and design. JL and HL: provision of study materials or patients and collection and assembly of data. JC, JL, KK, SYK, TL, K-MK, STK, and SJK: data analysis and interpretation. All authors: writing and final approval of manuscript.

### Conflict of Interest

SJK has served as consultant/advisor for Eli Lilly, Astellas, Foundation Medicine Inc., Pieris, Bristol Myers Squibb, Boston Biomedical and Merck. SJK has stock/equity in TP Therapeutics. JC has served as consultant/advisor for Eli Lilly, Merck, Boston Biomedical, and receives research funding (institutional) from Merck. The remaining authors declare that the research was conducted in the absence of any commercial or financial relationships that could be construed as a potential conflict of interest.
