# Supplementary material for: A Pilot Study of Baseline Spatial Genomic Heterogeneity in Primary Gastric Cancers Using Multi-Region Endoscopic Sampling
Source: Front Oncol. 2020 Feb 25;10:225. doi: 10.3389/fonc.2020.00225 (PMC7052337; doi:10.3389/fonc.2020.00225)
Supplement: Supplementary file 1 [file Table_1.DOCX]

**Supplemental Table 1.** Individual genes includes in the NGS analysis in patients undergoing multi-region endoscopic biopsy and cell-free DNA analyses.

| **Archer Reveal ctDNA kit 28 targets** | **Archer solid tumor kit 32 targets** |  | common |
| --- | --- | --- | --- |
| AKT1 | AKT1 |  | Cancer panel only |
| ALK | ALK |  | cfDNA only |
| BRAF | BRAF |  |  |
| CTNNB1 | CTNNB1 |  |  |
| DDR2 | DDR2 |  |  |
| EGFR | EGFR |  |  |
| ERBB2 | ERBB2 |  |  |
| FGFR1 | FGFR1 |  |  |
| HRAS | HRAS |  |  |
| IDH1 | IDH1 |  |  |
| IDH2 | IDH2 |  |  |
| KIT | KIT |  |  |
| KRAS | KRAS |  |  |
| MAP2K1 | MAP2K1 |  |  |
| MET | MET |  |  |
| PDGFRA | PDGFRA |  |  |
| PIK3CA | PIK3CA |  |  |
| RET | RET |  |  |
| ROS1 | ROS1 |  |  |
| TP53 | TP53 |  |  |
|  | CCND1 |  |  |
|  | EIF1AX |  |  |
|  | FGFR2 |  |  |
|  | FGFR3 |  |  |
|  | GNAS |  |  |
|  | MDM2 |  |  |
|  | NRAS |  |  |
|  | PTEN |  |  |
|  | RICTOR |  |  |
|  | STK11 |  |  |
|  | TERT |  |  |
|  | TSHR |  |  |
| ESR1 |  |  |  |
| AR |  |  |  |
| SMAD4 |  |  |  |
| NTRK3 |  |  |  |
| NTRK1 |  |  |  |
| MTOR |  |  |  |
| NRAS |  |  |  |
| MAP2K2 |  |  |  |
