# Supplementary material for: A Pilot Study of Baseline Spatial Genomic Heterogeneity in Primary Gastric Cancers Using Multi-Region Endoscopic Sampling
Source: Front Oncol. 2020 Feb 25;10:225. doi: 10.3389/fonc.2020.00225 (PMC7052337; doi:10.3389/fonc.2020.00225)
Supplement: Supplementary file 2 [file Table_2.DOCX]

**Supplementary Table 2.** DNA extraction results from multi-region endoscopic biopsy in 6 advanced gastric cancer cases included for sequencing analyses.

| **Tumor - biopsy region** | **case #** | **Conc. (ng/ul)** | **260/280** | **260/230** | **Total DNA(ug)** |
| --- | --- | --- | --- | --- | --- |
| tumor-1 | CASE I | 62.0 | 1.90 | 2.88 | 1.55 |
| tumor-2 | CASE I | 79.4 | 1.92 | 1.43 | 1.985 |
| tumor-3 | CASE I | 223.5 | 1.88 | 2.35 | 5.5875 |
| tumor-4 | CASE I | 81.6 | 1.89 | 2.24 | 2.04 |
| tumor-5 | CASE I | 88.2 | 1.91 | 2.06 | 2.205 |
| tumor-6 | CASE I | 60.8 | 1.90 | 2.34 | 1.52 |
| tumor-7 | CASE I | 109.3 | 1.89 | 2.24 | 2.7325 |
| tumor-1 | CASE II | 53.2 | 1.94 | 1.88 | 1.862 |
| tumor-2 | CASE II | 40.7 | 1.95 | 1.91 | 1.4245 |
| tumor-3 | CASE II | 65.9 | 1.94 | 2.05 | 2.3065 |
| tumor-4 | CASE II | 50.0 | 1.92 | 2.35 | 1.75 |
| tumor-5 | CASE II | 52.7 | 1.91 | 1.98 | 1.8445 |
| tumor-6 | CASE II | 40.5 | 2.00 | 1.92 | 1.4175 |
| tumor-7 | CASE II | 51.0 | 1.91 | 1.91 | 1.785 |
| tumor-8 | CASE II | 88.8 | 1.91 | 2.12 | 3.108 |
| tumor-1 | CASE III | 27.8 | 2.02 | 1.66 | 0.973 |
| tumor-4 | CASE III | 15.4 | 2.04 | 1.51 | 0.539 |
| tumor-5 | CASE III | 48.3 | 1.94 | 2.18 | 1.6905 |
| tumor-7 | CASE III | 41.5 | 1.96 | 1.88 | 1.4525 |
| tumor-8 | CASE III | 44.9 | 1.86 | 2.49 | 1.5715 |
| tumor-1 | CASE IV | 45.1 | 1.98 | 2.05 | 1.5785 |
| tumor-2 | CASE IV | 42.3 | 2.00 | 2.57 | 1.4805 |
| tumor-3 | CASE IV | 102.2 | 1.91 | 2.14 | 3.577 |
| tumor-4 | CASE IV | 52.7 | 1.94 | 2.60 | 1.8445 |
| tumor-5 | CASE IV | 104.7 | 1.91 | 2.30 | 3.6645 |
| tumor-6 | CASE IV | 64.0 | 1.94 | 2.15 | 2.24 |
| tumor-7 | CASE IV | 73.4 | 1.94 | 2.20 | 2.569 |
| tumor-8 | CASE IV | 61.9 | 1.97 | 2.38 | 2.1665 |
| tumor-1 | CASE V | 54.7 | 1.92 | 2.33 | 1.9145 |
| tumor-2 | CASE V | 64.5 | 1.94 | 2.39 | 2.2575 |
| tumor-3 | CASE V | 70.0 | 1.93 | 2.29 | 2.45 |
| tumor-4 | CASE V | 78.4 | 1.95 | 2.58 | 2.744 |
| tumor-5 | CASE V | 37.7 | 1.92 | 2.14 | 1.3195 |
| tumor-6 | CASE V | 52.6 | 1.95 | 2.42 | 1.841 |
| tumor-7 | CASE V | 52.8 | 1.94 | 2.16 | 1.848 |
| tumor-8 | CASE V | 51.8 | 1.92 | 1.93 | 1.813 |
| tumor-1 | CASE VI | 28.5 | 1.93 | 2.49 | 0.9405 |
| tumor-2 | CASE VI | 32.0 | 1.94 | 2.37 | 1.056 |
| tumor-3 | CASE VI | 32.2 | 1.92 | 2.21 | 1.0626 |
| tumor-4 | CASE VI | 31.1 | 1.98 | 1.82 | 1.0263 |
| tumor-5 | CASE VI | 14.7 | 2.01 | 1.58 | 0.4851 |
| tumor-6 | CASE VI | 26.2 | 1.94 | 2.05 | 0.8646 |
| tumor-7 | CASE VI | 30.1 | 1.96 | 1.61 | 0.9933 |
| tumor-8 | CASE VI | 37.7 | 1.93 | 2.15 | 1.2441 |
