# Supplementary material for: A Pilot Study of Baseline Spatial Genomic Heterogeneity in Primary Gastric Cancers Using Multi-Region Endoscopic Sampling
Source: Front Oncol. 2020 Feb 25;10:225. doi: 10.3389/fonc.2020.00225 (PMC7052337; doi:10.3389/fonc.2020.00225)
Supplement: Supplementary file 3 [file Data_Sheet_1.PDF]

VariantPlex® CTL

Part # SK0112

Description

The VariantPlex® CTL panel is an optimized balance of gene-specific primer (GSP) oligonucleotides that is used in conjunction with VariantPlex® HS Reagents for Illumina® (SK0117) and Molecular Barcode (MBC) Adapters to produce targeted NGS libraries of 290 regions of interest across 31 genes frequently mutated in NSCLC and thyroid cancer.

Contents

Description	Part Number	Storage Conditions
VariantPlex® CTL GSP1 - 8 reactions	SA0071081	-20°C ± 10°C
VariantPlex® CTL GSP2 - 8 reactions	SA0071082	
Archer® PreSeq™ DNA QC Assay Standard	SA0597	
Archer® PreSeq™ DNA QC Assay 10x Primer Mix	SA0598	

Required Reagent volumes:

Protocol Reference	Protocol Step*	Reagent	Required volume (per reaction)
A	Ligation Step 2 Elution	5mM NaOH	**36μL**
B	First PCR	VariantPlex® CTL GSP1 (SA0071081)	**4μL**
C	First PCR	10mM Tris-HCl pH 8.0	**38μL**
D	First PCR	Purified PCR1 eluate	**36μL**
E	Second PCR	VariantPlex® CTL GSP2 (SA0071082)	**4μL**

*Refers to steps within LA560

	Step	Temperature (°C)	Time	Cycles
**First PCR Reaction**	**1**	95	3 minutes	1
	**2**	95	30 seconds	18
	**3**	65	3 min (100% ramp rate)	
	**4**	72	3 minutes	1
	**5**	4	Hold	1
**Second PCR Reaction**	**1**	95	3 minutes	1
	**2**	95	30 seconds	24*
	**3**	65	3 min (100% ramp rate)	
	**4**	72	3 minutes	1
	**5**	4	Hold	1

Recommended PCR Cycling:

*The number of PCR2 cycles may be decreased if you regularly experience library yields greater than 200nM.

Recommended Reads and Multiplexing

VariantPlex CTL libraries produced should be sequenced to a minimum of **1.0M** reads per sample. Based on end-user experience, fewer reads may be sufficient for libraries prepared using limited input masses. For more information, visit our frequently asked questions resource page at: www.archerdx.com/faqs

Assay Targets

Gene	Accession Number	Exon Number	SNV & Small Indel	CNV
AKT1	NM_005163	3, 6	yes	no
ALK	NM_004304	21, 22, 23, 25	yes	yes
BRAF	NM_004333	11, 15	yes	no
CCND1	NM_053056	n/a (intergenic)	no	yes
CTNNB1	NM_001904	3	yes	no
DDR2	NM_006182	12, 13, 14, 15, 16, 17, 18	yes	yes
EGFR	NM_005228	3, 7, 15, 18, 19, 20, 21	yes	yes
EIF1AX	NM_001412	1, 2, 3, 4, 5, 6, 7	yes	no
ERBB2	NM_004448	10, 19, 20, 21, 24	yes	yes
FGFR1	NM_015850	4, 7, 8, 13, 15, 17	yes	yes
FGFR2	NM_000141	7, 9, 12, 14	yes	yes
FGFR3	NM_000142	7, 8, 9, 14, 15, 16, 18	yes	yes
GNAS	NM_000516	6, 7, 8, 9	yes	yes
HRAS	NM_005343	2, 3	yes	no
IDH1	NM_005896	3, 4	yes	no
IDH2	NM_002168	4	yes	no
KIT	NM_000222	2, 8, 9, 10, 11, 13, 14, 15, 17, 18	yes	yes
KRAS	NM_004985	2, 3, 4, 5	yes	yes
MAP2K1	NM_002755	2, 3	yes	no
MDM2	NM_002392	2, 3, 4, 6, 8, 10	no	yes
MET	NM_000245	2, 11, 14, 16, 19, 21	yes	yes
NRAS	NM_002524	2, 3, 4, 5	yes	yes
PDGFRA	NM_006206	12, 14, 15, 18, 23	yes	yes
PIK3CA	NM_006218	2, 5, 7, 8, 10, 14, 19, 21	yes	yes
PTEN	NM_000314	1, 2, 3, 4, 5, 6, 7, 8, 9	yes	yes
RET	NM_020630	10, 11, 13, 14, 15, 16	yes	yes
ROS1	NM_002944	38	yes	no
STK11	NM_000455	1, 2, 3, 4, 5, 6, 7, 8, 9	yes	yes
TERT	NM_198253	Promoter, 1	yes	no
TP53	NM_000546	1, 2, 3, 4, 5, 6, 7, 8, 9, 10, 11	yes	yes
TSHR	NM_000369	10	yes	no

Archer Analysis Settings

Sequencing data produced by this method must be converted to de-multiplexed FASTQ's, and then processed using **Archer Analysis** (v5.1 or greater). This provides all secondary analysis (read trimming/cleaning, de-duplication, error correction, alignment, and mutation calling), as well as some tertiary analysis (e.g., annotations and protein effect predictions). Analysis will produce detailed mutation reporting via graphical user interface, as well as raw text and BAM outputs.

The VariantPlex CTL libraries supports the following DNA Analysis Types: ***DNA Copy Number Variation, DNA SNP/InDel, and DNA Structural Variation*** in Archer Analysis (see the software user manual for further details on setting up analyses).

The Archer Analysis software is available as a separate download, which can be requested via a webform on the product webpage: Archer Analysis. VariantPlex CTL also requires a one-time upload of a Target Region file (a text file, in GTF format, which directs the software on how to analyze data from the panel) which can be obtained by contacting tech@archerdx.com.

Limitations of Use

For Research Use Only. Not for use in diagnostic procedures. Not intended to be used for treatment of human or animal diseases.

Safety data sheets pertaining to this product are available upon request.

© 2017 ArcherDX, Inc. All rights reserved. Reveal ctDNA™, VariantPlex®, PreSeq®, Archer® and FusionPlex® are trademarks of ArcherDX, Inc. Illumina®, NextSeq® and MiSeq® are registered trademarks of Illumina, Inc. Agencourt®, AMPure® and FormaPure® are registered trademarks of Agencourt Biosciences Corporation, a Beckman Coulter company. SYBR®, Life Technologies™, DynaMag™, Thermo Scientific™, Qubit™ and Maxima™ are registered trademarks of Thermo Fisher Scientific, Inc. KAPA Biosystems® is a registered trademark of KAPA Biosystems, Inc. RNase Away™ is a registered trademark of Molecular Bio-Products, Inc. BioRad®, iTaq™, and SsoAdvanced™ are registered trademarks of Bio-Rad Laboratories, Inc. Qiagen® and QuantiTect® are registered trademarks of Qiagen, Inc.

ArcherDX, Inc.
2477 55th Street, Suite 202
Boulder, CO 80301
303-357-9001
http://www.archerdx.com
