# Supplementary material for: A Pilot Study of Baseline Spatial Genomic Heterogeneity in Primary Gastric Cancers Using Multi-Region Endoscopic Sampling
Source: Front Oncol. 2020 Feb 25;10:225. doi: 10.3389/fonc.2020.00225 (PMC7052337; doi:10.3389/fonc.2020.00225)
Supplement: Supplementary file 4 [file Data_Sheet_2.PDF]

Reveal ctDNA™ 28

Part # SK0095, SK0106

Description

The Reveal ctDNA 28 panel is an optimized balance of gene-specific primer (GSP) oligonucleotides that is used in conjunction with Reveal ctDNA™ Reagents (SK0119) or VariantPlex® Somatic Reagents (SK0117) and Molecular Barcode (MBC) Adapters to produce targeted NGS libraries of 28 genes frequently mutated in solid tumor type cancers.

Contents

Description	Part Number	Storage Conditions
Reveal ctDNA™ 28 GSP1 - 8 reactions	SA0095081	-20°C ± 10°C
Reveal ctDNA™ 28 GSP2 - 8 reactions	SA0095082	

Required Reagent volumes:

Protocol Reference	Protocol Step	Reagent	Required volume (per reaction)
A	Ligation Step 2 Elution	5mM NaOH	**36µL**
B	Step 5: First PCR	Reveal ctDNA™ 28 GSP1 (SA0095081)	**4µL**
C	Step 5: First PCR	10mM Tris-HCl pH 8.0	**38µL**
D	Step 5: First PCR	Purified PCR1 eluate	**36µL**
E	Step 6: Second PCR	Reveal ctDNA™ 28 GSP2 (SA0095082)	**4µL**

Recommended PCR Cycling:

	Step	Temperature (°C)	Time	Cycles
**First PCR Reaction**	**1**	95	3 minutes	1
	**2**	95	30 seconds	15
	**3**	65	5 min (100% ramp rate)	
	**4**	72	3 minutes	1
	**5**	4	Hold	1
**Second PCR Reaction**	**1**	95	3 minutes	1
	**2**	95	30 seconds	15-18*
	**3**	65	5 min (100% ramp rate)	
	**4**	72	3 minutes	1
	**5**	4	Hold	1

*Use 18 cycles for total sample input masses <9ng. The number of PCR2 cycles may be decreased if you regularly experience library yields greater than 200nM.

Recommended Reads and Multiplexing

Analysis of Reveal ctDNA 28 libraries produced with Reveal ctDNA Reagents requires a minimum of **5M** reads per sample, while libraries produced with VariantPlex Reagents require a minimum of **1M** reads per sample. Based on end-user experience, fewer reads may be sufficient for libraries prepared using limited input masses. For more information, visit our frequently asked questions resource page at: www.archerdx.com/faqs

Assay Targets

Gene	Accession	Target Exon
ALK	NM_004304	22,23,25
AKT1	NM_005163	3
AR	NM_033031	4,5,8
BRAF	NM_004333	11,15
CTNNB1	NM_001904	3
DDR2	NM_006182	17
EGFR	NM_005228	12,18,19,20,21
ERBB2	NM_004448	8,20
ESR1	NM_000125	5,7,8
FGFR1	NM_015850	13
HRAS	NM_005343	2,3
IDH1	NM_005896	4
IDH2	NM_002168	4
KIT	NM_000222	9,11,13,17,18
KRAS	NM_004985	2,3,4
MAP2K1	NM_002755	2,3
MAP2K2	NM_030662	3
MET	NM_000245	14
NRAS	NM_002524	2,3
NTRK1	NM_002529	14,15
NTRK3	NM_002530	16,17
PIK3CA	NM_006218	10,21
PDGFRA	NM_006206	12,14,16,18
RET	NM_020630	11,13,14,15,16
ROS1	NM_002944	38,40
SMAD4	NM_005359	9
MTOR	NM_004958	44,45,50
TP53	NM_000546	Full exon

Archer Analysis Settings

Sequencing data produced by this method must be converted to de-multiplexed FASTQ's, and then processed using **Archer Analysis** (v5.0 or greater). This provides all secondary analysis (read trimming/cleaning, de-duplication, error correction, alignment, and mutation calling), as well as some tertiary analysis (e.g., annotations and protein effect predictions). Analysis will produce detailed mutation reporting via graphical user interface, as well as raw text and BAM outputs.

The Reveal ctDNA 28 libraries produced with Reveal ctDNA reagents require selection of the ***cfDNA*** pipeline found within the ***DNA SNP/InDel*** Analysis Type in Archer Analysis (see the software user manual for further details on setting up analyses). This target enrichment panel does not support detection of chimeric gene fusions. The cfDNA pipeline includes two mechanisms to expedite and customize the interpretation process:

1. The default filter set for the Variant Grid (found in the Variant Summary tab) is optimized to screen out variants that are of low statistical confidence or simply not of interest – this filter set can and should be further customized to suit end-user specific assay requirements.
2. A Targeted Mutation file is essential for identifying known variants of interest at low allele fractions. This is a tab-delimited text file in Variant Call Format (VCF), which is included in the analysis set up process. An example Targeted Mutation file is included with the Reveal ctDNA™ 28 panel, which contains over 1,000 known driver mutations (most found in COSMIC) captured by the Assay Targets listed in the table above. This file is a great starting point, but should be customized according to the end users' specific needs.

NOTE: If using VariantPlex reagents, be sure to select the ***somatic DNA*** pipeline instead and manually turn on error correction in the Advanced tab of the Perform Analysis page.

The Archer Analysis software is available as a separate download, which can be requested via a webform on the product webpage: Archer Analysis. Reveal ctDNA™ 28 also requires a one-time upload of a Target Region file (a text file, in GTF format, which directs the software on how to analyze data from the panel). The optional Target Mutation file discussed above also requires a one-time upload. Both of these files can be obtained by contacting tech@archerdx.com.

Limitations of Use

For Research Use Only. Not for use in diagnostic procedures. Not intended to be used for treatment of human or animal diseases.

Safety data sheets pertaining to this product are available upon request.

© 2017 ArcherDX, Inc. All rights reserved. Reveal ctDNA™, VariantPlex®, PreSeq®, Archer® and FusionPlex® are trademarks of ArcherDX, Inc. Illumina®, NextSeq® and MiSeq® are registered trademarks of Illumina, Inc. Agencourt®, AMPure® and FormaPure® are registered trademarks of Agencourt Biosciences Corporation, a Beckman Coulter company. SYBR®, Life Technologies™, DynaMag™, Thermo Scientific™, Ion Torrent™, Qubit™ and Maxima™ are registered trademarks of Thermo Fisher Scientific, Inc. KAPA Biosystems® is a registered trademark of KAPA Biosystems, Inc. RNase Away™ is a registered trademark of Molecular Bio-Products, Inc. BioRad®, iTaq™, and SsoAdvanced™ are registered trademarks of Bio-Rad Laboratories, Inc. Qiagen® and QuantiTect® are registered trademarks of Qiagen, Inc.

ArcherDX, Inc.
2477 55th Street, Suite 202
Boulder, CO 80301
303-357-9001
http://www.archerdx.com
